# Spatial Variation in Mercury Accumulation in Bottlenose Dolphins (*Tursiops* spp.) in Southeastern U.S.A.

**DOI:** 10.3390/toxics12050327

**Published:** 2024-04-30

**Authors:** Mackenzie L. Griffin, Colleen E. Bryan, Tara M. Cox, Brian C. Balmer, Russell D. Day, Laura Garcia Barcia, Antoinette M. Gorgone, Jeremy J. Kiszka, Jenny A. Litz, Robin M. Perrtree, Teri K. Rowles, Lori H. Schwacke, Randall S. Wells, Eric Zolman

**Affiliations:** 1 Department of Marine and Environmental Sciences, Savannah State University, Savannah, GA 31404, USA; mackenzie.griffin@noaa.gov (M.L.G.); coxt@savannahstate.edu (T.M.C.); perrtreer@savannahstate.edu (R.M.P.); 2 Chemical Sciences Division, National Institute of Standards and Technology, Charleston, SC 29412, USA; rusty.day@manta-online.org; 3 Conservation Medicine, National Marine Mammal Foundation, Charleston, SC 29405, USA; bcbalmer1@gmail.com (B.C.B.); lschwacke@mmc.gov (L.H.S.); eric.zolman@nmmpfoundation.org (E.Z.); 4 Marine Science and Nautical Training Academy, Charleston, SC 29412, USA; 5 Institute of Environment, Department of Biological Sciences, Florida International University, Biscayne Bay Campus, Miami, FL 33199, USAjkiszka@fiu.edu (J.J.K.); 6 Marine Mammal and Turtle Division, Southeast Fisheries Science Center, National Marine Fisheries Service, National Oceanic and Atmospheric Administration, Miami, FL 33149, USA; annie.gorgone@noaa.gov (A.M.G.); jenny.litz@noaa.gov (J.A.L.); 7 Cooperative Institute for Marine and Atmospheric Studies, University of Miami, Miami, FL 33149, USA; 8 Marine Mammal Health and Stranding Response Program, National Marine Fisheries Service, National Oceanic and Atmospheric Administration, Silver Spring, MD 20910, USA; teri.rowles@noaa.gov; 9 Marine Mammal Commission, Bethesda, MD 20814, USA; 10 Sarasota Dolphin Research Program, Brookfield Zoo Chicago, c/o Mote Marine Laboratory, Sarasota, FL 34236, USA; rwells@mote.org

**Keywords:** total mercury (THg), bottlenose dolphin, sentinel species, cetacean, marine mammal

## Abstract

Bottlenose dolphins (*Tursiops* spp.) inhabit bays, sounds, and estuaries (BSEs) throughout the southeast region of the U.S.A. and are sentinel species for human and ecosystem-level health. Dolphins are vulnerable to the bioaccumulation of contaminants through the coastal food chain because they are high-level predators. Currently, there is limited information on the spatial dynamics of mercury accumulation in these dolphins. Total mercury (THg) was measured in dolphin skin from multiple populations across the U.S. Southeast Atlantic and Gulf of Mexico coasts, and the influence of geographic origin, sex, and age class was investigated. Mercury varied significantly among sampling sites and was greatest in dolphins in St. Joseph Bay, Florida Everglades, and Choctawhatchee Bay (14,193 ng/g ± 2196 ng/g, 10,916 ng/g ± 1532 ng/g, and 7333 ng/g ± 1405 ng/g wet mass (wm), respectively) and lowest in dolphins in Charleston and Skidaway River Estuary (509 ng/g ± 32.1 ng/g and 530 ng/g ± 58.4 ng/g wm, respectively). Spatial mercury patterns were consistent regardless of sex or age class. Bottlenose dolphin mercury exposure can effectively represent regional trends and reflect large-scale atmospheric mercury input and local biogeochemical processes. As a sentinel species, the bottlenose dolphin data presented here can direct future studies to evaluate mercury exposure to human residents in St. Joseph Bay, Choctawhatchee Bay, and Florida Coastal Everglades, as well as additional sites with similar geographical, oceanographic, or anthropogenic parameters. These data may also inform state and federal authorities that establish fish consumption advisories to determine if residents in these locales are at heightened risk for mercury toxicity.

## 1. Introduction

Bottlenose dolphins (*Tursiops* spp.) are sentinels for human and ecosystem-level health due to their high degree of site fidelity, long lifespan, and high trophic level, including consuming some of the same diet items as humans [1,2,3]. As such, the bioaccumulation of contaminants in dolphin tissues can reflect localized sources of pollution [1,4]. Bottlenose dolphins can reflect some of the greatest levels of bioaccumulation seen among wildlife [1,5,6,7]. Bottlenose dolphins along the coasts of the southeast U.S.A. that inhabit bays, sounds, and estuaries (BSEs) include two closely related species, Tamanend’s bottlenose dolphins (*Tursiops erebennus*) and common bottlenose dolphins (*Tursiops truncatus*), which were recently genetically delineated [8].

The main transport of mercury into marine ecosystems is through atmospheric deposition and surface runoff [9,10,11,12]. Inorganic mercury is methylated into methylmercury (MeHg) by sulfate-reducing bacteria and biomagnified through the food web via dietary consumption [12,13,14,15,16,17]. The rate of methylation and MeHg release from sediments increases with temperature, salinity, and the availability of organic carbon [12,18]. Methylmercury is the most toxic and most common form of organic mercury in the environment and can cause deleterious health effects in both humans and dolphins [12,19,20]. Mercury toxicity in humans can cause diminished cardiovascular health, endocrine disruption, motor and sensory abnormalities, visual and hearing deficits, and fetal abnormalities [21,22]. Similarly, elevated mercury levels in dolphins and other organisms have been correlated with negative effects on the hepatic, renal, and endocrine systems [23]. Bottlenose dolphins from the Indian River Lagoon (IRL), along the east coast of Florida, have four times greater mercury exposure than bottlenose dolphins in Charleston, South Carolina (CHS), and human residents within IRL have elevated mercury exposure relative to the CHS reference population [24]. Residents who consumed most of their seafood from local recreational sources had significantly greater mercury in their hair than those who consumed seafood that was not from local waters [24]. The elevated mercury exposure of both bottlenose dolphins and humans further demonstrates the suitability of dolphins as a sentinel species for human and public health [24]. Mercury exposure in bottlenose dolphins can be attributed to atmospheric deposition from anthropogenic activities (i.e., coal combustion, industrial uses, waste incineration, and mining), low freshwater input, or low tidal flushing in the area of residency. Low freshwater input can result in increased salinity, and greater salinity increases mercury binding to sediments with the potential mobilization of mercury [25,26]. Enclosed bays can experience minimal tidal flushing, facilitating the accumulation of mercury [25]. In the Florida Coastal Everglades (FCE), one cause of mercury deposition hotspots is the slow water movement and greater dissolved organic carbon that enhances methylation [27,28].

The goal of this study was to provide a comprehensive assessment of mercury in free-ranging bottlenose dolphins throughout BSEs in the southeast region of the U.S.A. and investigate the spatial variation in mercury exposure in bottlenose dolphins throughout the region. Dolphin skin tissue was used to quantify baseline mercury in individuals and evaluate possible relationships between mercury exposure and biological factors, such as sex and age.

## 2. Materials and Methods

*Sampling locations.* This study encompasses BSE bottlenose dolphin sample sets measured for mercury from Georgia and Florida and published data sets from South Carolina and Florida [7,28,29,30] (Table 1). Sampling sites measured for mercury in Georgia BSEs included the Skidaway River Estuary (SRE), Sapelo Island (SAP), and Brunswick (BRU); Florida BSEs included Biscayne Bay (BBF), St. Joseph Bay (SJB), and Choctawhatchee Bay (CBF). Sites with previously published data included Charleston, SC (CHS), Indian River Lagoon, FL (IRL), Florida Coastal Everglades (FCE), Lower Florida Keys (LFK), and Sarasota Bay, FL (SAR).

*Sample collection.* Population monitoring efforts during skin sample collection can use dorsal fin features and photo-identification to identify individual dolphins to track life history events, fine-scale habitat use, and health outcomes throughout their lives [31,32]. Skin samples from individual dolphins were used as a proxy for overall exposure because mercury levels in the skin have a significant positive correlation to mercury measured in internal organs, including the muscle, liver, and kidney [7,30,33,34]. Dolphin skin samples were collected from 2005 to 2019 via catch-and-release health assessments [1,35], remote biopsy [25,36], or both. Skin samples were placed in a cryovial and frozen until analysis. Additional sample collection details are in the Supplementary Materials. Several individual dolphins were sampled more than once (determined via genetic and/or photo-identification matches). In turn, the mercury value from the most recently collected sample was used in data analysis.

The sex of the dolphins was determined using molecular methods for samples collected via remote biopsy [37] and physical examination for dolphins sampled during catch-and-release health assessments. Lower Florida Keys and CBF dolphins were excluded from the analysis of the influence of sex on exposure due to low sample sizes with determined sex (*n* < 5). The ages of dolphins sampled during catch-and-release health assessments were estimated by postnatal dentine layers from an extracted tooth [35,38] or known birth year. When a tooth was not available for aging or the birth year was not known, age class was estimated based on the measurement of the total animal length. Age classes were defined as calf < 2 years or <200 cm in length; subadult ≥ 2 years and <10 years or ≥200 cm and <240 cm; adult ≥ 10 years or ≥240 cm [35]. For SRE dolphins sampled by remote dart biopsy, only female adults were identified from reproductive history data via photo-identification and sighting history. Biscayne Bay, LFK, and CBF were excluded from the age class comparison because this information was not available.

Mercury analysis. Total mercury (THg) measured herein is a proxy for the toxic form, methylmercury (MeHg), in cetacean skin since most (89% to 97%) of the mercury in the skin is MeHg [30,33]. Before mercury analysis, any residual blubber was trimmed from the skin sample. Mercury measurement methods varied among sites because mercury analyses were conducted by several of the coauthors (Table 1). However, mercury measurement results utilizing isotope dilution cold vapor inductively coupled plasma mass spectrometry (ID-CV-ICP-MS) [39,40], atomic fluorescence spectroscopy (AFS) [7,41,42], and direct combustion atomic absorption spectrometry (DC AAS) [43,44,45] were fully comparable among analytical methods and validated using reference materials [46]. For further details on mercury analysis methods and control materials, see the Supplementary Materials. Mercury was measured on a wet mass (nanogram per gram mass fraction) basis. All measurements are reported in ng/g wet mass (wm) THg and are referred to as mercury in the rest of this paper.

**Figure 1:**
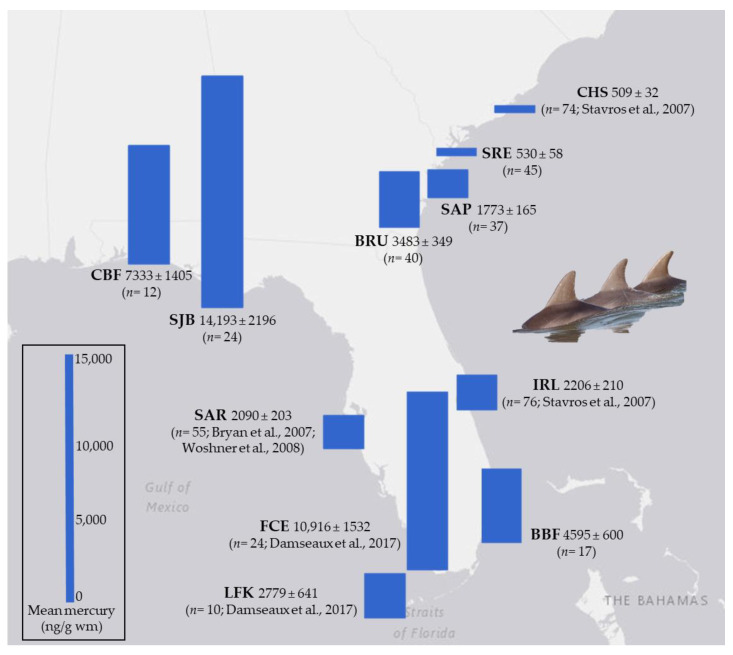
Mean mercury (ng/g, wet mass) in skin from bottlenose dolphins (*Tursiops* spp.) sampled along the U.S. Southeast Atlantic and Gulf of Mexico coasts. Sampling sites included Charleston (CHS) [29], Skidaway River Estuary (SRE), Sapelo Island (SAP), Brunswick (BRU), Indian River Lagoon (IRL) [29], Biscayne Bay (BBF), Florida Coastal Everglades (FCE) [28], Lower Florida Keys (LFK) [28], Sarasota Bay (SAR) [7,30], St. Joseph Bay (SJB), and Choctawhatchee Bay (CBF) The number of dolphins sampled within each site is represented as *n*.

*Statistical analyses.* The authors from previously published studies [7,28,29,30] shared individual animal mercury and life history (sex, age) data for statistical comparison among all dolphin BSE sample sites in this study (Figure 1; Table 1). All mercury data were tested for normality using the Shapiro–Wilk test. Data that failed the normality test were log-transformed prior to statistical analyses to meet the assumptions of normality and equal variance. A full factorial analysis of variance (ANOVA) was used to analyze the relationship of mercury exposure to the collection site, sex, and age class for dolphins (RStudio 1.2.5003). A Tukey’s honest significance test was used to determine significant differences between the factor levels (*p* < 0.05).

## 3. Results and Discussions

### 3.1. Spatial Variations 

**Figure 2:**
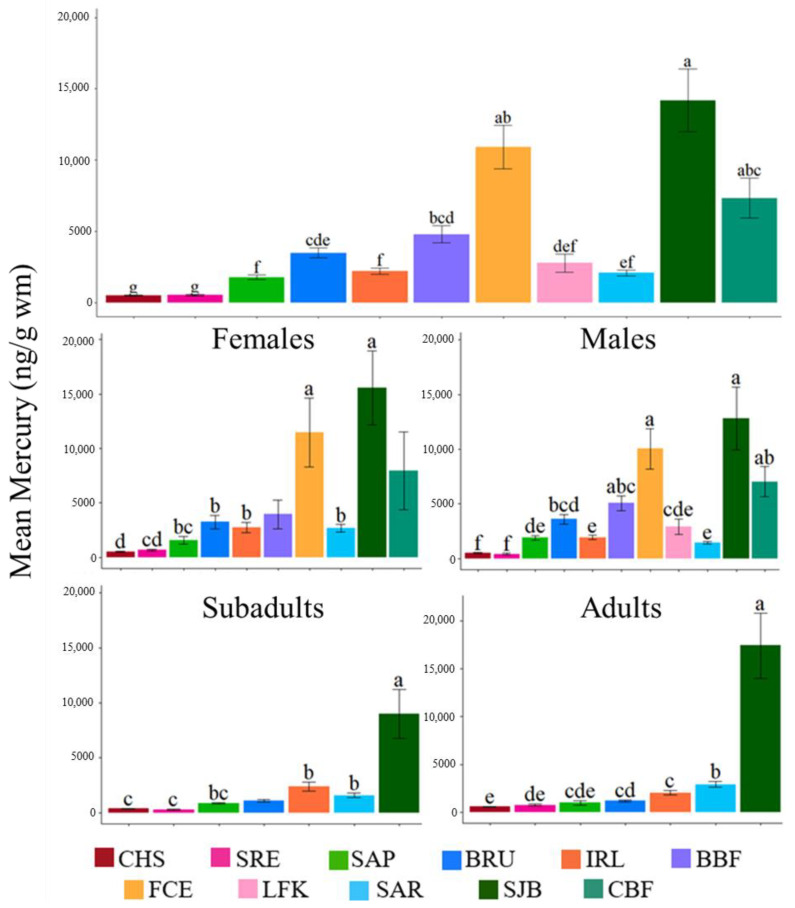
Mean mercury ± SE (ng/g, wet mass) in skin from bottlenose dolphins (*Tursiops* spp.) sampled along the U.S. Southeast Atlantic and Gulf of Mexico coasts. Sampling sites included Charleston (CHS), Skidaway River Estuary (SRE), Sapelo Island (SAP), Brunswick (BRU), Indian River Lagoon (IRL), Biscayne Bay (BBF), Florida Coastal Everglades (FCE), Lower Florida Keys (LFK), Sarasota Bay (SAR), St. Joseph Bay (SJB), and Choctawhatchee Bay (CBF). The mean mercury of all dolphin individuals at each site is displayed in the top graph. Sites with different letters above the error bars are significantly different (*p* < 0.05) from each other.

We measured mercury in 175 individual dolphin skin samples from six sites along the U.S. Southeast Atlantic and Gulf of Mexico coasts (Table 2). These individuals were then compared with published data from 239 dolphins (a grand total of 414 dolphins). Mercury in dolphins was significantly higher [*F* (10, 403) = 72.41, *p* < 0.05] in SJB, FCE, and CBF and significantly lower in CHS and SRE (Figure 2). Mercury in dolphins from CHS and SRE was significantly lower [*F* (10, 403) = 72.41, *p* < 0.05] than at all other sites but not significantly different from each other (*p* = 1; Figure 2). This spatial pattern was also found among different sexes and age classes (Table 3 and Table 4).

Greater mercury exposure in dolphins can be attributed to locality, anthropogenic activities, methylation rates, low freshwater input, and/or low tidal flushing. Additionally, the distribution of mercury within estuaries can be influenced by the tidal regime [47,48,49]. The Port of St. Joseph Bay has a history of paper mill operations that produced mercury waste. From 1938 to 1974, operators discharged mill wastewater into an unlined impoundment, which went directly into nearby wetland areas and SJB [50,51]. Consequently, low levels of mercury were found in soil samples from SJB [50,51]. Also, SJB is an enclosed bay dominated by *Spartina* sp. salt marshes and has low tidal energy [52]. *Spartina* can distribute mercury by accumulating within the root system, transferring it to the leaf tissue, and subsequently, environmental release through hydathodes (salt-excreting organs) [53,54,55]. While this movement of mercury from the *Spartina* root system to above-ground tissues is limited [56], in combination with the anthropogenic source and the fact that it is an enclosed bay, could have caused elevated mercury exposure within SJB.

Greater mercury exposure within the FCE is likely due to the natural biogeochemical processes of mangrove forests [28]. Unlike *Spartina*, mercury readily adsorbs onto the surface of mangrove sediment particles with organic matter and dissolved organic carbon, which is then tidally pumped into the surrounding waters [27]. In addition, mangrove mud is acidic (pH 3 to 4) and promotes mercury availability for anaerobic bacteria that are developed from higher organic content and enable methylation [28].

Similar to SJB, CBF is a *Spartina* sp. dominated saltmarsh ecosystem with untreated stormwater runoff, agricultural activities, and municipal wastewater that flows into the Choctawhatchee watershed [52]. Choctawhatchee Bay is also a largely enclosed embayment with low tidal energy that is not substantially influenced by freshwater inflow; this combination of factors may allow for greater mercury accumulation within the bay [25,52,57]. Altogether, mercury exposure in dolphins was greatest in SJB and CBF, likely due to anthropogenic sources and low freshwater input, and in the FCE as a result of natural biological and geochemical processes.

Mercury in dolphins from CHS and SRE was significantly lower [*F* (10, 403) = 72.41, *p* < 0.05] than at all other sites but not significantly different from each other (*p* = 1; Figure 2). Charleston Harbor has an active shipping channel and coal-burning power plants located within the watershed, which may contribute to mercury in the coastal environment. However, there is high freshwater flow and tidal flushing that may result in low mercury exposure to the dolphins [58]. Dolphins within CHS may have low mercury exposure due to increased freshwater flow from the nearby river basins and an effective tidal exchange that carries mercury out of the harbor and into the Atlantic Ocean.

Similarly, SRE has high freshwater input and large tidal flushing in addition to relatively low anthropogenic sources of mercury. The SRE is part of the relatively unpolluted Ogeechee River basin [59]. The nearest power plant to the Ogeechee River basin is 22.5 km east of the headwaters of the basin [60]. This power plant shut down coal-burning operations in 2016, and the predominant wind direction is from the west and south; therefore, atmospheric mercury may be carried away from the watershed [60]. Additionally, SRE has the maximum southern Atlantic tidal amplitude range of 2.1 m [61], which may be transporting mercury out of the estuary and into the Atlantic Ocean.

The mean mercury exposure in dolphin skin from SAP, BRU, IRL, BBF, LFK, and SAR was significantly higher than CHS and SRE (Figure 2). Additionally, SAP, IRL, LFK, and SAR were all significantly lower than SJB, FCE, and CBF. See Figure 2 for the statistical mercury relationship among sites. There are some potential anthropogenic sources causing the chronic exposure of mercury to the dolphins in these areas, but not acute and primary sources. Brunswick was the only site with known and confirmed elevated mercury exposure due to an anthropogenic source. In BRU, there are four Superfund sites resulting in the surrounding marsh being severely contaminated by metals and organics from contaminated waste, which is improperly discharged into the watershed [62]. One of the Superfund sites, LCP Chemicals, operated as a chlor-alkali plant from the 1956s to 1994 and is a source for mercury and other contaminants within BRU. LCP Chemicals discharged over 1 kg of inorganic mercury daily for approximately 40 years, along with other contaminants, into the nearby estuaries, and high levels of residual mercury still exist today [62,63,64,65,66,67]. Sapelo Island is the closest sampling site to BRU and may be receiving some mercury from the LCP Chemicals site despite SAP being 30 km north of BRU and within a sparsely populated National Estuarine Research Reserve [68,69,70]. Similarly, BBF has a natural mangrove shoreline and shares the watershed with FCE due to its close proximity. Overall, SAP, IRL, BBF, LFK, and SAR had varying amounts of tidal ranges and flushing with potential sources for the mercury effluent, but there was no clear pattern of mercury exposure to the dolphins in these BSEs.

### 3.2. Influence of Sex and Age

Skidaway River Estuary and SAR were the only two of eight sites that had a significant difference in mercury within each site between female and male dolphins. Skidaway River Estuary and SAR female dolphins had significantly higher mercury levels than males within the same site (*F* (1, 43) = 9.733, *p* = 0.003 and *F* (1, 53) = 6.343, *p* = 0.01, respectively)(Table 2). Charleston, SRE, and SAR were three of six sites where adults had significantly higher mercury levels than subadults (*F* (1, 72) = 10.03, *p* = 0.002; *F* (1, 21) = 20.23, *p* < 0.001; and *F* (1, 47) = 18.5, *p* < 0.001, respectively) (Table 3). When comparing sex and age class with mercury exposure within the same site, significant differences were found in CHS and SAR but not IRL. Female adults in CHS had significantly higher mercury levels than male adults (*F* (3, 70) = 6.48, *p* = 0.048) and female subadults (*p* < 0.001), but not significantly higher levels than male subadults (*p* = 0.075). Female adults in SAR had significantly higher mercury levels than all other SAR dolphins (*F* (3, 45) = 12.9, *p* < 0.001).

Age and sex can influence mercury levels in individual dolphins. Dolphins are considered a “closed system” because they lack hair and surface glands, and mercury is primarily obtained through their diet with limited capabilities of excretion [13,14,15,16,41]. Adult female dolphins may have increased mercury exposure compared to males due to increased prey consumption during gestation and lactation to fulfill energy requirements [71]. Mercury is only minimally offloaded to the offspring through placental transfer or lactation [72,73,74,75]. Adult dolphins are likely to have greater mercury exposure compared to younger age classes because mercury is also significantly positively correlated with the age of dolphins due to the continued consumption of prey and lack of significant mercury excretion [7,29,30,76]. For sites not exhibiting significant differences between sexes, it may be that females and males are consuming prey at a similar rate because there may be a lack of actively reproducing females or that males are continuing to grow, whereas females reach an asymptotic length by approximately 10 y to 12 y [77,78]. Similarly, there may not be a significant difference in mercury levels between subadults and adults because subadults may be consuming more prey than adults to maintain energy demands for growth, thus masking significant differences among age classes. Along the central west coast of Florida, subadults have altered their foraging and ranging patterns in response to environmental changes and prey availability [79,80]. Lastly, age determination for individuals may differ from fine-scale differences in animal size and sexual maturity. However, regardless of sex or age class, mercury spatial patterns were consistent geographically.

## 4. Conclusions

Dolphins serve as sentinel species for human and ecosystem health, and analyzing mercury via skin samples can help identify localized sources of mercury contamination in their environment over time [1,2,4]. The mean residence time for mercury in the ocean is from 20 y to 30 y [12], and spatial comparison for mercury may identify the most vulnerable and heavily affected areas, where dolphins, humans, and other high trophic level organisms might be at greatest risk from exposure [12]. This was the first study to compare mercury exposure in dolphin populations along the U.S. Southeast Atlantic and Gulf of Mexico coasts. Mercury exposure is driven by a complex mix of biogeographical characteristics in the study areas and the anthropogenic activities in these areas. Regardless of sex or age class, spatial patterns were significant and consistent. Dolphins in St. Joseph Bay, Choctawhatchee Bay, and the Florida Coastal Everglades had significantly greater mean mercury exposure than most sites; dolphins in Charleston and Skidaway River Estuary had significantly less exposure. Dolphins can effectively represent regional trends and reflect large-scale atmospheric mercury input and local biogeochemical processes. Dolphins are sentinels for human health, and this study informs state and federal authorities evaluating mercury exposure to human residents in St. Joseph Bay, Choctawhatchee Bay, and Florida Coastal Everglades, as well as additional sites with similar geographical, oceanographic, or anthropogenic parameters to determine if residents in these locales are at heightened risk for mercury toxicity.

## Figures and Tables

**Table 1:** Summary of bottlenose dolphins (*Tursiops* spp.) sampled along the U.S. Southeast Atlantic and Gulf of Mexico coasts. Sampling sites included Charleston (CHS), Skidaway River Estuary (SRE), Sapelo Island (SAP), Brunswick (BRU), Indian River Lagoon (IRL), Biscayne Bay (BBF), Florida Coastal Everglades (FCE), Lower Florida Keys (LFK), Sarasota Bay (SAR), St. Joseph Bay (SJB), and Choctawhatchee Bay (CBF). Information includes the year(s) sampled, number of individuals (*n*), sample collection method, and mercury analysis method. Sample collection methods included isotope dilution cold vapor inductively coupled plasma mass spectrometry (ID-CV-ICP-MS), atomic fluorescence spectroscopy (AFS), and direct combustion atomic absorption spectrometry (DC AAS).

Site	Year	*n*	Sample Collection	Mercury Analysis
CHS [29]	2003–2005	74	Catch-and-release	DC AAS
SRE	2015 & 2017	45	Remote biopsy	DC AAS
SAP	2007–2009	37	Remote biopsy and catch-and-release	ID-CV-ICP-MS
BRU	2006–2007 and 2009	40	Remote biopsy and catch-and-release	ID-CV-ICP-MS
IRL [29]	2003–2005	76	Catch-and-release	DC AAS
BBF	2019	17	Remote biopsy	AFS
FCE [28]	2013	24	Remote biopsy	DC AAS
LFK [28]	2008	10	Remote biopsy	DC AAS
SAR [7,30]	2002–2005	55	Catch-and-release	AFS
SJB	2005–2006	24	Catch-and-release	ID-CV-ICP-MS
CBF	2007	12	Remote biopsy	ID-CV-ICP-MS

**Table 2:** Mean mercury ± standard error (SE) (ng/g, wet mass) in skin from bottlenose dolphins (*Tursiops* spp.) sampled along the U.S. Southeast Atlantic and Gulf of Mexico coasts. Sampling sites included Charleston (CHS), Skidaway River Estuary (SRE), Sapelo Island (SAP), Brunswick (BRU), Indian River Lagoon (IRL), Biscayne Bay (BBF), Florida Coastal Everglades (FCE), Lower Florida Keys (LFK), Sarasota Bay (SAR), St. Joseph Bay (SJB), and Choctawhatchee Bay (CBF). Sites with fewer than five individuals were excluded from the sex analysis and labeled NE (not examined). * Sites with significant differences (*p* < 0.05) between female and male mercury levels (ng/g, wm).

Site	Total *n*	Hg (ng/g, wm)	Female *n*	Female Hg (ng/g, wm)	Male *n*	Male Hg (ng/g, wm)	Unknown Sex *n*
CHS [29]	74	509 ± 32	29	509 ± 52	45	509 ± 42	0
SRE *	45	530 ± 58	18	687 ± 92	27	425 ± 70	0
SAP	37	1773 ± 165	11	1570 ± 338	25	1894 ± 192	1
BRU	40	3483 ± 349	14	3257 ± 621	26	3605 ± 428	0
IRL [29]	76	2206 ± 210	25	2756 ± 477	51	1936 ± 202	0
BBF	17	4595 ± 600	4	3949± 1313	13	5058 ± 684	0
FCE [28]	24	10,916 ± 1532	8	11,460 ± 3156	13	10,048 ± 1841	3
LFK [28]	10	2779 ± 641	0	NE	9	2936 ± 694	1
SAR [7,30] *	55	2090 ± 203	29	2665 ± 343	26	1448 ± 98	0
SJB	24	14,193 ± 2196	12	15,585 ± 3400	12	12,802 ± 2874	0
CBF	12	7333 ± 1405	4	7949 ± 3570	8	7024 ± 1373	0

**Table 3:** Mean mercury ± standard error (SE) (ng/g, wet mass) in skin from bottlenose dolphins (*Tursiops* spp.) sampled along the U.S. Southeast Atlantic and Gulf of Mexico coasts by age class. Sampling sites included Charleston (CHS), Skidaway River Estuary (SRE), Sapelo Island (SAP), Brunswick (BRU), Indian River Lagoon (IRL), Sarasota Bay (SAR), and St. Joseph Bay (SJB). Sites with fewer than five individuals were excluded from age analysis and labeled NE (not examined). * Sites with significant differences (*p* < 0.05) between subadult and adult mercury values (ng/g, wm).

Site	Calf *n*	Calf Hg (ng/g, wm)	Subadult *n*	Subadult Hg (ng/g, wm)	Adult *n*	Adult Hg (ng/g, wm)
CHS [29] *	0	NE	25	391 ± 33	49	568 ± 44
SRE *	0	NE	8	291 ± 43	15	758 ± 100
SAP	1	597	5	882 ± 73	8	978 ± 243
BRU	0	NE	3	1107 ± 92	9	1172 ± 126
IRL [29]	0	NE	30	2377 ± 410	45	2039 ± 221
SAR [7,30] *	6	662 ± 99	24	1592 ± 210	25	2910 ± 323
SJB	2	10,540 ± 892	7	9010 ± 2235	14	17,407 ± 3389

**Table 4:** Mean mercury ± standard error (SE) (ng/g, wet mass) in skin from bottlenose dolphins (*Tursiops* spp.) sampled along the U.S. Southeast Atlantic and Gulf of Mexico coasts by sex and age class. Sampling sites included Charleston (CHS), Skidaway River Estuary (SRE), Sapelo Island (SAP), Brunswick (BRU), Indian River Lagoon (IRL), Sarasota Bay (SAR), and St. Joseph Bay (SJB). Sites with less than five individuals were excluded from sex and age analysis. * Sites with significant differences (*p* < 0.05) mercury (ng/g, wm) among sex and age class values.

Site	Calf		Subadult		Adult	
	*n*	Hg (ng/g, wm)	*n*	Hg (ng/g, wm)	*n*	Hg (ng/g, wm)
**CHS** [29] *						
Female	0		17	363 ± 38.5	12	716 ± 81.1
Male	0		8	452 ± 61.8	37	521 ± 48.7
**SRE**						
Female	0		1	254	15	758 ± 100
Male	0		7	296 ± 48.9	0	
**SAP**						
Female	1	597	4	934 ± 66.7	2	843 ± 170
Male	0		1	674	6	1023 ± 326
**BRU**						
Female	0		3	1107 ± 91.7	2	1652 ± 176
Male	0		0		7	1035 ± 108
**IRL** [29]						
Female	0		12	2984 ± 796	12	2378 ± 597
Male	0		18	1972 ± 426	33	1916 ± 214
**SAR** [7,30] *						
Female	4	683 ± 109	9	1866 ± 524	16	3609 ± 408
Male	2	619 ± 270	15	1428 ± 129	9	1666 ± 104
**SJB**						
Female	1	9648	5	10,919 ± 2701	5	21,995 ± 7152
Male	1	11,431	2	4238 ± 86.0	9	14,857 ± 3543

## Data Availability

Data are available from the corresponding author on request.

## Supplementary Materials

The following supporting information can be downloaded at: https://www.mdpi.com/article/10.3390/toxics12050327/s1, The following supporting information includes additional method details for the remote biopsy and catch-and-release sampling, and mercury analyses.
