# Supplementary material for: Spatial Variation in Mercury Accumulation in Bottlenose Dolphins (Tursiops spp.) in Southeastern U.S.A
Source: Toxics. 2024 Apr 30;12(5):327. doi: 10.3390/toxics12050327 (PMC11125612; doi:10.3390/toxics12050327)
Supplement: Supplementary file 1 [file toxics-12-00327-s001.zip › toxics-2920902-supplementary.pdf]

Supporting Information

Common Bottlenose Dolphin Skin Sample Collection

Remote Biopsy Sampling

Remote biopsy sampling was done following methods described in Sinclair et al., 2015[36] and Balmer et al., 2019[25]. A crossbow was used with untethered bolts to collect remote biopsy samples from common bottlenose dolphins at each site. Prior to biopsy sampling, all bolts, tips, and subsampling gear were cleaned and/or sterilized[25,36]. Pre-cleaned 25 mm stainless steel sampling tips (10 mm internal diameter) were threaded on to bolts outfitted with an integrated float. The sampling tips had an externally beveled sharpened cutting edge and internal prongs angled down to retain the sample once collected from the targeted animal. Sampling distance was 2 to 10 m and ideal biopsy tip placement was the flank of the dolphin below the dorsal fin and above the midline[82], perpendicular to the sampler, so the tip impacted the dolphin as flush as possible. For all biopsy shots, a photographer attempted to collect images of sampling location on the body, sampled individual's dorsal fin, and reaction to biopsy sampling. Post-shot, the floating bolt was retrieved by hand from the water's surface. If the shot resulted in a hit, the bolt was retired for the day whether a sample was collected or not to avoid any cross-contamination of samples until the bolt and tip were completely cleaned and/or sterilized. If the shot missed, the bolt was reused if it only contacted water. The bolt was retrieved, the shaft dried, and the sampling tip rinsed with ethanol prior to reloading. If there was any question whether the bolt struck the target dolphin, it was not reused until completely cleaned and/or sterilized to avoid possible cross-contamination with a subsequent sample. Following a successful remote biopsy attempt, the sample consisted of skin and a full depth section of blubber. The sample was removed from the cutterhead tip using solvent-rinsed utensils and placed onto a Teflon sheet.

Subsamples were processed and prepared for storage immediately. The skin was dissected from the blubber and subsampled for different analyses. Mercury skin samples were placed in a 2 mL cryovial and then frozen at ultra cold temperatures (-80 ºC or below) until analysis.

<u>*Catch-and-Release Sampling*</u>

Catch-and-release sampling for wedge biopsies was done following methods described in Wells et al., 2004[1] and Wells et al., 2005[31]. Schwacke et al., 2010[35] details CBF and SJB and Schwacke et al., 2012[83] details BRU and SAP catch-and-release health assessment sample collection. Small groups or individual bottlenose dolphin were selected and encircled with a seine net to capture in shallow waters. Once an animal is restrained by handlers, the sex is determined, and females are examined by ultrasound to determine reproductive status. Pregnant females had modified in-water examination and sampling, which did not include wedge biopsy collection. Non-pregnant females and males were individually brought aboard a veterinary examination boat for measurements, examination, and sample collection. A wedge biopsy of skin and full depth blubber (approximately 4 cm long x 3 cm wide x 1.5 cm deep) was removed under local anesthesia using solvent cleaned and autoclaved surgical instruments and the site was cleaned with antiseptic prior to removal. The biopsy site was approximately 10 cm posterior and 10 cm ventral from the caudal insertion of the dorsal fin. The skin was dissected from the blubber and both tissues were subsampled for various analyses. Mercury skin samples were placed in 2 mL cryovials, stored in the field in a liquid nitrogen vapor-phase shipper, and long-term frozen at ultra cold temperatures (-80 ºC or below) until analysis.

Mercury Analysis

AFS Methods

Total mercury mass fractions in dart biopsy skin samples from BBF bottlenose dolphins were measured by atomic fluorescence spectrometry (AFS) following a Standard Operating Procedure modified from the U.S. Environmental Protection Agency (EPA) Test Method 1631[84] and Garcia Barcia et al., 2020[43] and external calibration. The external calibration curves were prepared using Mercury AA Standard (Lab Chemand Ricca Chemical Company, TX). Skin samples were hot block digested using digestion tubes with nitric acid (HNO_3) for two hours of gradual heating to 120 °C, followed by the addition of hydrogen peroxide (H_2O_2) and diluted using 1 % hydrochloric acid (HCl) before AFS analysis. In addition to the skin samples and controls, procedural blanks were carried through the entire sample processing and measurement scheme. The Hg mass fractions in procedural blanks were not subtracted from skin and control samples since all procedural blanks were below the method detection limit. DORM-4 Fish Protein Certified Reference Material for Trace Metals (NRC Canada) was used as a control material and measured with the skin samples for method validation and quality control. Measured Hg values for DORM-4 were in agreement with certified Hg values.

DC AAS Methods

Total mercury mass fractions in dart biopsy skin samples from SRE bottlenose dolphins were measured by direct combustion atomic absorption spectrometry (DC AAS) with a direct mercury analyzer using external calibration. The external calibration curves were prepared by gravimetrically aliquoting different masses of aqueous dilutions of NIST SRM 3133 Mercury Standard Solution (Lot 14 No. 160921) into quartz sample boats. All bottlenose dolphin skin

samples were weighed directly into nickel weigh boats and placed on the auto-sampler rotor with control materials and procedural blanks (clean empty nickel weigh boat). The mean Hg mass fraction in procedural blanks was subtracted from the mass fraction of Hg in each skin and control sample. NIST SRM 1946 Lake Superior Fish Tissue (Gaithersburg, MD) was used as a control material and measured with the skin samples for method validation and quality control. Measured Hg values for SRM 1946 were in agreement with certified Hg values.

ID-CV-ICP-MS Methods

Total mercury mass fraction in dart biopsy and catch-and-release skin samples from BRU, CBF, SAP, and SJB bottlenose dolphins were measured by isotope dilution cold vapor inductively coupled plasma mass spectrometry (ID-CV-ICP-MS). Skin samples were microwave digested in glass vessels with HNO_3 and H_2O_2. The samples were spiked with ^{201}Hg enriched isotopic spike (National Isotope Development Center, Oak Ridge, TN). Prior to use, the ^{201}Hg isotopic spike was calibrated by reverse isotope dilution using NIST SRM 3133 Mercury Standard Solution (Lot No. 991304; Gaithersburg, MD) and NIST SRM 1641d Mercury in Water (Gaithersburg, MD). Digested samples were diluted to approximately 0.5 ng/g ^{201}Hg using high purity water with 2 % HNO_3 and 2 % HCl. Mercury measurements were made using cold-vapor Hg generation coupled with ICP-MS isotope ratio measurements. The Hg vapor was generated using tin (II) chloride reductant (10 % mass fraction in 7 % volume fraction HCl), separated from the liquid phase using a glass gas-liquid separator cell, and transferred to the ICP-MS. The ^{201}Hg and ^{202}Hg isotopes were monitored to calculate the Hg measured in skin, control material, and procedural blank samples. In addition to the skin samples and controls, procedural blanks, containing a small aliquot of ^{201}Hg spike only, were carried through the entire sample processing and measurement scheme. The mean Hg (ng) in procedural blanks was subtracted from ng of Hg

in each skin and control sample. DORM-2 Dogfish Muscle Certified Reference Material for Trace Metals (NRC Canada) and QC05-SKN01C (NIST in-house cryo-homogenized bottlenose dolphin skin control material) were used as control materials and measured with the skin samples for method validation and quality control. The measured control Hg values fell within the expanded uncertainty of their certificate values.
